# Remote or in-clinic? The effect of service delivery mode on hearing aid output: study protocol for a double-blinded, randomised trial in adults with mild to moderate sensorineural hearing loss

**DOI:** 10.1186/s13063-024-08068-y

**Published:** 2024-04-12

**Authors:** Craig Lett, David Welch, Rosie Dobson

**Affiliations:** School of Population Health, University of Auckland, Auckland, New Zealand

**Keywords:** Teleaudiology, Audiology, Hearing aids, Rehabilitation, Health service delivery, Telehealth

## Abstract

**Background:**

Teleaudiology can potentially improve access to hearing healthcare services. Remote hearing aid fittings offer a new mode of service delivery that removes barriers of geography and access to an audiologist. Real-ear measurements (REMs) are the gold standard for hearing aid output verification but require in-clinic appointments. This study will investigate whether remote hearing aid fittings can provide clinically equivalent outcomes when compared to current, in-clinic, best practice guidelines.

**Research design:**

A repeated measure, double-blinded crossover design will be used. Participants will be randomly allocated to one of two groups to determine order of intervention, balanced for degree of hearing loss.

**Study sample:**

Sixty adults with mild to moderate hearing loss and at least 1 year of experience with hearing aids will be recruited.

**Data collection and analysis:**

Participants will complete two hearing aid fitting protocols, one using an in-clinic fitting process and the other using a remote (at-home) fitting process. In-clinic fittings will include REMs with adjustments to standard (NAL-NL2) prescription targets. The two fitting protocols will then be randomly assigned to participants in a crossover design, so participants and researchers will be blinded to the order of the two fitting protocols. Participants will then have a 4-week period with follow-up appointments for participant-directed gain adjustment. For each fitting protocol, participants will complete objective measurements of final hearing aid output with REMs, speech-in-noise testing, subjective measurements of hearing aid performance, and quality of life measurements. They will then begin an identical period of living with, adjusting, and objective assessment with the other fitting protocol. Data will be analysed as repeated measures with statistical control for potential confounding variables.

**Results:**

Data will compare the four-frequency average real-ear aided response (4FREAR) for hearing aids programmed in-clinic and hearing aids programmed remotely, after participant-directed gain adjustments. Secondary measures will assess clinically significant differences in estimated speech intelligibility, hearing-related quality of life, hearing aid benefit, sound quality and preference, and speech-in-noise ability.

**Conclusions:**

This study will inform the development of best practice guidelines for remote hearing aid fittings. If no clinically significant differences are found between in-clinic and remote fit hearing aids, it has the potential to expand teleaudiology initiatives.

**Trial registration:**

Australian New Zealand Clinical Trial Registry, ACTRN12623000028606p. Date of registration: 12 January 2023.

**Supplementary Information:**

The online version contains supplementary material available at 10.1186/s13063-024-08068-y.

## Introduction

### Background and rationale {6a}

The World Health Organization estimates 1 in 4 people will have some degree of hearing loss by 2050, with 1 in 10 having a disabling hearing loss [1]. There is a global challenge in providing access to quality hearing care services, with low-income countries particularly affected [1]. Even among high-income countries, hearing care services are not universally accessible. It is estimated that there is approximately one audiologist per 17,000 people in the UK [2] and approximately one per 7000 people in New Zealand.

Teleaudiology is one way to increase access to hearing care services by overcoming geographic barriers and potentially decreasing costs, both of which are important for addressing the needs of underserved populations [3–5]. However, teleaudiology is also part of a paradigm shift in the provision of hearing healthcare services. It can offer a more person-centred approach, where the consumer can be more engaged in the rehabilitation process [6]. Teleaudiology has the potential to significantly improve hearing healthcare globally, but continued research is still needed to ensure strong, evidence-based care [7].

Hearing aids have used Bluetooth technology since 2005, with the Starkey ELI, but it was the launch of the GN Resound LiNX made-for-iPhone hearing aid in 2014 that paved the way for modern teleaudiology. Hearing aid manufacturers can now use apps and Bluetooth technology to allow an audiologist to connect to a person’s hearing aids directly through a smartphone. The audiologist has almost complete access to make remote changes to the hearing aid settings.

Audiology regulatory bodies have responded by creating basic guidelines for teleaudiology, confirming that it is an appropriate model of service delivery when the quality of service and patients’ outcomes are equal to those provided by traditional in-clinic services [8–10]. Multiple studies have confirmed teleaudiology as a valid method of service delivery [11–13]. While Covid-related restrictions led to a rise in the use of teleaudiology, a significant number of barriers remain [14–17]. Continued research into patient outcomes and best practice is required to increase the uptake of teleaudiology by audiologists as an appropriate service model.

Self-reported outcomes are reported to be the same for people who had an in-clinic hearing aid fitting and follow-up process and those who had remote follow-up appointments [18], but it is important to also consider objective clinical measures. Furthermore, Tao et al. included a facilitator, present in the subject’s home, to assist the remote audiologist, with a laptop to connect to the hearing aid and provide a video and audio link. More recent updates in hearing aid technology allow audiologists to use individual’s smartphones for a video and audio link as well as connecting directly to the individual’s hearing aids via apps (for example, myPhonak by Phonak, TeleHear by Starkey Hearing Technologies and Smart 3D by GN ReSound). This negates the need for a facilitator and requires the individual to take a more active role in the appointment.

In New Zealand, the best practice guidelines for tuning hearing aids is to adjust the output to prescribed targets (most commonly the National Acoustics Laboratory Non-Linear 2 prescription (NAL-NL2)), verified with objective clinic measurements that assess the hearing aid output by frequency across multiple signal input levels, adjusted to the ear canal acoustics of the individual [19]. This measurement can be performed in a test box or as a real-ear measurement (REM) with the hearing aids positioned in the individual’s ear canal. A real-ear aided response (REAR) is obtained, which is a measurement of the sound levels in the ear canal accounting for the input signal, the individual ear canal acoustics and the hearing aid amplification. Audiologists adjust the output of the hearing aid until the REAR matches prescribed targets. The New Zealand Audiological Society allows a tolerance from the prescribed targets of + / − 5 dB for frequencies up to 2000 Hz and + / − 8 dB for 3000 and 4000 Hz [19]. In practice, audiologists also use a person’s subjective report to adjust hearing aid settings: comments such as “tinniness” or “sharpness” may lead to decreases in high-frequency amplification; reports of cutlery and crockery being unbearably “clattery” may lead to overall reduction in volume in quieter environments. Hearing aids programmed using manufacturer’s initial fit without REMs show statistically poorer self-reported listening ability and listening in noise, but the effect is small and it is not clear whether there is a clinically significant benefit [20].

The auditory system is highly adaptive, and an individual’s hearing will begin to change in response to the increased sound levels they experience upon the initial fitting. As a result, the audiologist will typically interact closely with each person over time until an optimal listening point is arrived at. With audiologists and individuals working closely to finalise hearing aid settings over multiple appointments, REMs could be considered as a starting point; the final hearing aid settings may vary greatly from the initial measured setting, in order to meet expectations and needs, and to achieve a successful hearing aid outcome.

Teleaudiology allows the audiologist to use the person’s real-world environment to assist with hearing aid programming. A person could be sitting in their kitchen or living room during an appointment, allowing the audiologist to make environment-specific changes to the hearing aid settings which can immediately be tested and re-tuned as needed. This should allow audiologists to get to appropriate settings much more quickly, and also much more specifically. Through teleaudiology, an audiologist may achieve the same final hearing aid output settings with purely remote sessions as they would with in-clinic sessions.

Once an individual uses telehealth they will often continue using telehealth in the future [21], but for audiologists to feel confident in recommending a telehealth option research needs to show that optimal subjective and objective outcomes can be met or that they can be met to a degree that is not inferior to an in-clinic alternative.

### Objectives {7}

Teleaudiology allows hearing aids to be programmed and adjusted remotely but to date no studies have compared objective measures from a fully remotely programmed hearing aid with those of an in-clinic programmed hearing aid. The purpose of this study is to compare gain output and patient outcomes for hearing aids programmed remotely to those programmed in-clinic to assist in the development of well-defined, standardised protocols for clinical practice.

### Trial design {8}

This protocol describes a non-inferiority, double-blind randomised crossover trial to evaluate the effectiveness of using teleaudiology for the programming of hearing aids. Outcomes are measured 4 weeks after each intervention, with a 2-week period between interventions. The study will take 14 weeks for each participant.

This protocol is in accord with the Standard Protocol Items: Recommendations for Interventional Trials (SPIRIT) 2013 statement [22], and the intervention is described according to the Consolidated Standards of Reporting Trials (CONSORT) checklist [23]. See Supplementary material for the complete SPIRIT checklist.

## Methods: participants, interventions and outcomes

### Setting {9}

In-clinic appointments will take place at the University of Auckland Hearing and Tinnitus Clinic or in a private audiology clinic in the Auckland region. Remote appointments will connect to the home of each participant (or a location of their preference). Participants will be asked to choose a quiet location where they were unlikely to be interrupted for the duration of the appointment.

### Eligibility criteria {10}

People eligible for the study must comply with all of the following at time of enrolment:

1. Diagnosed sensorineural hearing loss between mild and moderate, according to WHO definitions [1], with thresholds unchanged within ± 10 dB over the past 12 months. Participants with complex hearing needs (tinnitus, single-sided deafness, conductive loss greater than 15 dB) are excluded from this study;
2. At least 1 years’ experience as a hearing aid wearer. Experienced hearing aid users are familiar with the basic functions of hearing aids (switching on and off, insertion and removal) required for remote fitting services. Experienced hearing aid users have higher and more stable sound output requirements than new hearing aid users;
3. Access to a compatible smartphone or tablet with connection to the internet in a suitably private location (min 5 Mb upload/download speed). Participants will be given the use of an appropriate smartphone if they do not have a compatible one themselves.
4. Available for the duration of the study and to attend in-clinic appointments.

### Who will take informed consent? {26a}

Informed consent will be obtained by trained clinical research assistants and/or a clinician at the start of the first appointment and before any data is collected.

### Additional consent provisions for collection and use of participant data and biological specimens {26b}

Not applicable. No biological specimens will be collected.

## Interventions

### Explanation for the choice of comparators {6b}

In-clinic hearing aid programming with real-ear verification is the current gold standard for audiology in New Zealand and many other countries. Remote hearing aid fitting provides a useful alternative, providing clinical outcomes are equal. The intervention protocols have been developed to replicate real-world processes in order to identify any clinically significant differences.

### Intervention description {11a}

**Fig. 1:**
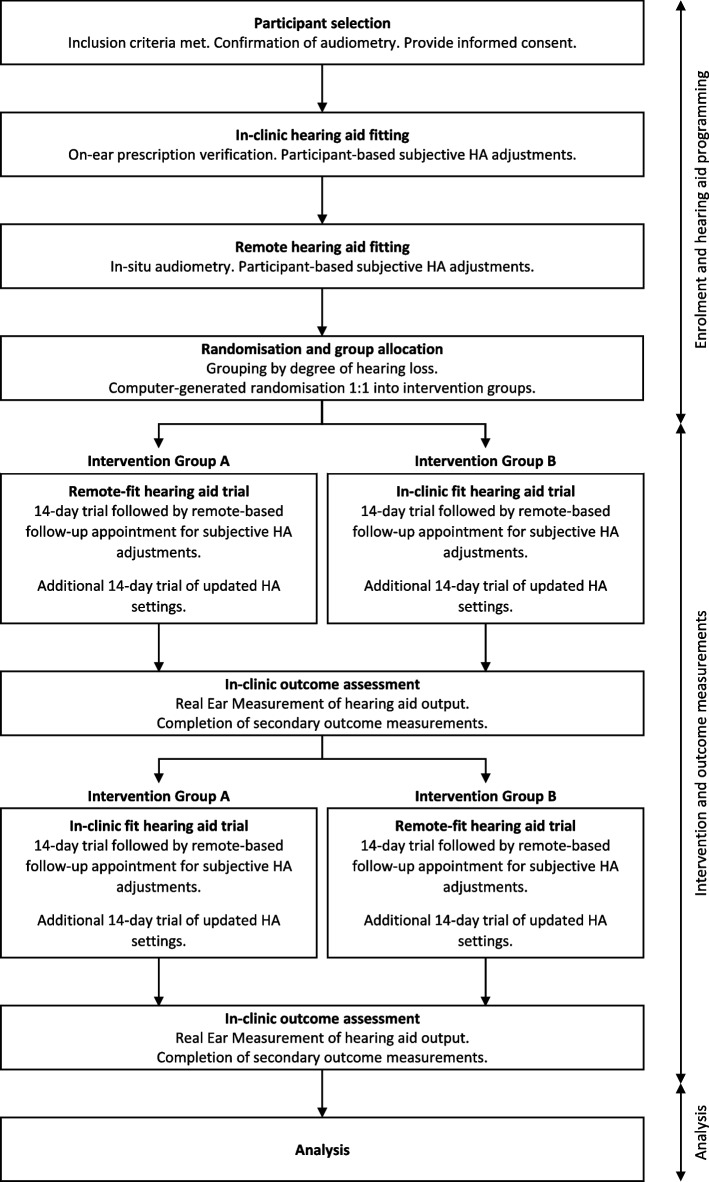
Study flow

The study flow is presented in Fig. 1. Interested participants will be contacted to discuss the study, go through the Participant Information Sheet, answer questions and schedule the first appointment. Consent will be obtained at the start of the first appointment and before any data is collected.

Participants will complete a standard audiometry test battery (pure-tone audiometry for air- and bone-conduction thresholds, speech audiometry, and immittance audiometry) in accordance with the New Zealand Audiological Society Best Practice Guidelines [24–26] in order to confirm study selection criteria are met and to ensure accurate hearing aid prescription.

Participants will have two hearing aid fittings: an in-clinic hearing aid fitting with real-ear verification and a remote hearing aid fitting with subjective verification.

Participants will be given the opportunity to go into a prize draw in acknowledgement and thanks for their time and participation in the study. While the hearing aids used in the study will be returned at the conclusion of the study, participants will have the opportunity to privately purchase new hearing aids of the same model and performance if they would like to do so.

#### Initial in-clinic hearing aid fitting

For the initial in-clinic hearing aid fitting, participants will be fit binaurally with premium-level, rechargeable, receiver-in-the-canal hearing aids. The receiver and dome configuration will be chosen using standard clinical procedures and be consistent for each participant for the duration of the study. The hearing aids will be programmed through the manufacturer’s software to 100% acclimatisation using the National Acoustics Laboratory Non-Linear 2 (NAL-NL2) prescription targets for long-term hearing aid wearers.

REAR will be verified using a Natus Aurical Freefit and Otometrics Otosuite PMM module, with the gain settings adjusted through hearing aid software to meet NAL-NL2 targets for soft (55 dB), medium (65 dB), and loud (75 dB) stimuli using the International Speech Test Signal. On-ear measurement of maximum power output will be measured at 85 dB to ensure estimated uncomfortable loudness levels are not exceeded.

Where hearing aid programming features are available for an in-clinic session but are not available for a remote session, these will be removed from the in-clinic fitting protocol to maintain blinding for the clinicians performing the follow-up appointments.

Hearing aid settings will be optimised using subjective participant feedback to balance overall volume, perceived sound quality, and perception of own voice/occlusion. This process will follow an interview-style approach, with the participant asked to comment on each component and the clinician making small adjustments to the hearing aid settings until the participant is satisfied with the sound. The hearing aid setting will then be stored and the hearing aids retained at the clinic.

Participants will be required to have an app installed on their smartphone or tablet before the remote hearing aid fitting appointment. They will be guided through this process at the end of the initial in-clinic fitting appointment.

#### Initial remote hearing aid fitting

Approximately 2 weeks after the in-clinic hearing aid fitting, a remote hearing aid fitting appointment will take place. The time interval is to allow participants’ memory of the sound of the in-clinic programming to decay so that it is less likely to influence the remote fitting. The same hearing aids used at the in-clinic hearing aid fitting will be reset in-clinic and programmed through hearing aid software to 100% acclimatisation using NAL-NL2 prescription targets for long-term hearing aid wearers. The hearing aids will be programmed to allow for a remote hearing aid connection.

The hearing aids will be sent to the participant via courier.

At the scheduled hearing aid fitting appointment time, the clinician will telephone the participant to confirm the app installation and Bluetooth pairing was successful. The participant and clinician will then connect through the app for a remote support session.

Remote support allows an audio–video link between the participant and clinician. The clinician will connect to the participant’s hearing aids through a Bluetooth connection with the participant’s smartphone or tablet.

When both the participant and the clinician are connected to the remote support appointment, the clinician will perform proxy verification measurements through the hearing aid manufacturer software.

Hearing aid settings will be optimised using subjective participant feedback to balance overall volume, perceived sound quality, and perception of own voice/occlusion. This process will follow an interview-style approach, with the participant asked to comment on each component and the clinician making small adjustments to the hearing aid settings until the participant is happy with the sound.

The hearing aid settings will be saved, and the participant will return the hearing aids to the clinic through return-post courier.

#### Group allocation

Participants will be randomly assigned to one of two groups (“Group A” and “Group B”). Group A will test the remotely fit hearing aids first, followed by the in-clinic fit hearing aids. Group B will test the in-clinic fit hearing aids first, followed by the remotely fit hearing aids. The participants will be blind to group and order of hearing aids, as will the clinician performing follow-up appointments and assessments.

#### First and second interventions

The hearing aids will be programmed with the pre-saved settings that relate to each participant’s group allocation. Two weeks after the final fitting appointment, the participants will be sent the hearing aids via courier. They will be instructed to wear them for at least 8 h per day for 2 weeks.

At the end of these 2 weeks, all participants will have a follow-up appointment via remote support with a clinician who is blinded to the groups. At this appointment, the participants will have the opportunity to have the hearing aid settings adjusted by the clinician in order to optimise the sound for their personal preferences. This process will follow an interview-style approach, with the participant asked to comment on overall volume, overall sound quality, performance in quiet, performance in noise, and perception of own voice. The clinician will make small adjustments to the hearing aid settings until the participant is satisfied with the sound.

Participants will be instructed to wear the hearing aids for a further 2 weeks, and then return to the clinic for the first outcome assessment. The hearing aids will be returned to the clinic at this time.

The second intervention will follow the same process as the first, with the hearing aids programmed with the alternate settings.

### Criteria for discontinuing or modifying allocated interventions {11b}

Interventions will not be modified during the study. Participants will remain on the study unless they withdraw themselves.

### Strategies to improve adherence to interventions {11c}

Adherence can be monitored at 2-week intervals through hearing aid data-logging software that includes use-tracking data.

### Relevant concomitant care permitted or prohibited during the trial {11d}

Participants will wear the provided hearing aids during the study in place of their existing hearing aids.

### Provisions for post-trial care {30}

At the end of the intervention period, participants will return to their existing hearing aids under the care of their personal audiologist.

### Outcomes {12}

Participants will attend an in-clinic appointment to measure outcomes. Outcome measurements will occur in weeks 8 and 14 (4 weeks after the start of each intervention period). The purpose of the crossover design is to overcome the order effects, so the outcomes for the two time points will be combined within the treatment groups.

#### Primary outcome

The primary outcome is the absolute difference between the 4FREAR for each intervention.

The clinician will measure the real-ear aided response of each hearing aid following the NZAS Best Practice Guidelines using a Natus Aurical Freefit and Otometrics Otosuite PMM module for soft (55 dB), medium (65 dB), and loud (75 dB) signal outputs using the International Speech Test Signal, and an on-ear Maximum Power Output measurement at 85 dB [19].

#### Secondary outcomes

Secondary outcome measures include:

- *Estimated speech intelligibility* measured by the Speech Intelligibility Index (SII) of each hearing aid fitting. An SII of zero indicates no intelligible speech and a score of one indicates all speech information is available to the participant. The REAR allows an SII to be calculated for each signal strength, allowing for comparison between interventions.
- *Hearing-related quality of life* measured using the Hearing Handicap Inventory for Adults (HHIA), a 25-item questionnaire that measures hearing-related QOL across social and emotional subdomains [27].
- *Hearing aid benefit* measured using the Abbreviated Profile of Hearing Aid Benefit (APHAB), a 24-item questionnaire that measures hearing aid benefit across four subscales: ease of communication (EC), reverberation (RV), background noise (BN), and aversiveness (AV)
- *Sound quality and preference* measured on 5-point Likert scales. Participants are asked to rate the overall sound quality of each intervention, and rate their preference between the interventions.
- *Process acceptability* (how likely the participant is to recommend the process to another person) measured as a net promotor score for each intervention.
- *Speech-in-noise ability* measured for each intervention with an unaided and aided quick speech-in-noise (QSIN) test. QSIN will be performed according to the QSIN user guide in a calibrated testing room with speech and background noise (four-speaker babble) presented from a single speaker in front of the participant at a level of 65 dB HL. A QSIN score for each intervention will be calculated from the average SNR loss across 3 standard test lists.

### Participant timeline {13}

**Fig. 2:**
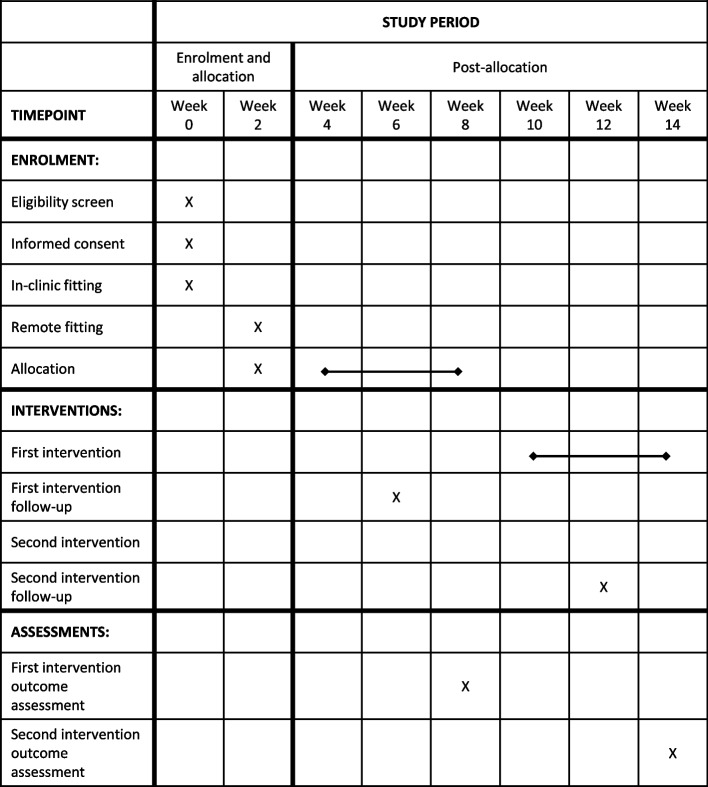
Schedule of enrolments, interventions and assessments

Figure 2 shows the schedule of enrolment, interventions, and assessments.

### Sample size {14}

Sixty participants (thirty per group) will be recruited for the trial. This will provide 90% power at the 5% significance level to detect an overall clinically meaningful difference of 5 dB 4FREAR and allow for up to 10% attrition.

Power analysis was conducted by selecting a random sample of patients from a clinical register and using the variance in REM scores to estimate the expected variance in the research. Power analysis for a 5-dB non-inferiority limit (the acceptable variance in REMs according to the NZAS Best Practice Guidelines) in a continuous outcome in a non-inferiority trial with an alpha level of 5% and 90% power indicates a total sample size of 54 (27 per group). We propose a sample size of 60, which will allow 30 in each hearing loss group and therefore a repeated measures analysis on groups of 30.

### Recruitment {15}

Recruitment will take place through the University of Auckland Hearing and Tinnitus Clinic and through private audiology clinics in the Auckland region. Recruitment will be via flyers and posters in clinic and on social media (Facebook and Instagram) networks. Posters will also be disseminated through audiologist networks so that they can invite people to participate in the study.

## Assignment of interventions: allocation

### Sequence generation {16a}

Eligible participants will be grouped according to degree of hearing loss (mild and moderate, according to WHO classifications [1]) to ensure an even spread of hearing levels between intervention groups. Participants within each hearing loss group will be assigned a random number as per a computer-generated randomisation schedule, then allocated to group A or B in the order ABAB, with an allocation ratio of 1:1, by a clinical research assistant.

### Concealment mechanism {16b}

Participants are assigned to one of the intervention groups but the group is concealed from them. They will not be aware of which fitting method they are trialling at any stage. Clinicians are not aware of which groups the participants have been assigned. A research assistant will allocate participants and will deidentify the hearing aid fitting files within Noah software. Each participant will have 2 hearing aid files (intervention A and B), and it will not be apparent to the researcher which intervention setting is being used at any time. The researcher will not know which group a participant is allocated by the research assistance until after the research is complete, at which point the research assistant will share the electronic records.

### Implementation {16c}

All participants who meet the eligibility criteria and complete the in-clinic and remote hearing aid fitting process will be randomised and assigned to an intervention group by a trained clinical research assistant. The research assistant will generate the allocation sequence.

## Assignment of interventions: blinding

### Who will be blinded {17a}

This is a double-blind study. Both the participants and the clinicians will be blind to the allocation of groups and order of intervention. The hearing aid settings and real-ear measurements will be stored anonymously so that the clinician is unable to determine what fitting process was used. The participants and clinicians will be aware of the intervention strategy at the fitting process (in-clinic or remote), which occurs prior to group allocation and blinding.

### Procedure for unblinding if necessary {17b}

There is no requirement for unblinding.

## Data collection and management

### Plans for assessment and collection of outcomes {18a}

Primary and secondary outcome measurements will be assessed 4 weeks after each intervention at an in-clinic appointment.

### Plans to promote participant retention and complete follow-up {18b}

All participant appointments will be confirmed 2 weeks and 48 h prior to each appointment. All outcome assessments will be completed in person.

### Data management {19}

Data will be managed through REDCap.

### Confidentiality {27}

Data will be maintained electronically on a secure server at the University of Auckland. The information collected from participants will remain confidential and will be deidentified for analysis purposes.

### Plans for collection, laboratory evaluation, and storage of biological specimens for genetic or molecular analysis in this trial/future use {33}

Not applicable for this study as there are no biological specimens.

## Statistical methods

### Statistical methods for primary and secondary outcomes {20a}

The difference in REAR measures between interventions will be used as the primary outcome. The data will be modelled as a fully factorialised 5-way mixed-measures ANOVA with repeated measured being: ear (right and left), frequency (500, 1000, 2000, 4000 Hz), REM signal input level (55 dB, 65 dB, 75 dB), and fitting method (in-clinic or remote); and level of hearing loss (mild or moderate) as a between-participant factor.

Secondary analyses of speech intelligibility, hearing-related quality of life, hearing aid benefit, sound quality and preference, and speech-in-noise ability will be conducted in the same way. In the analyses, a main or interaction effect involving fitting method will provide evidence that the in-clinic and remote fitting approaches led to differences in the variables involved. Interactions will be explored using testing of simple effects using follow-up ANOVAs and graphical representations of data including error bars based on the standard error of the mean difference between the points, to represent the repeated measured nature of these variables, and using the standard error of the mean for analysis related to level of hearing loss.

Given the statistical power inherent in the data analysis (repeated measures approach), the sample size should be sufficient. To ascertain the clinical importance of the difference observed at a statistical level, a criterion of + / − 5 dB will be applied to REAR measures, in accordance with best practice guidelines. The secondary outcomes do not have a convention associated with clinical importance, so any statistical effects observed will be discussed in terms of the specific measure and meaningful outcomes in previous research as well as making comparisons based on confidence intervals.

### Interim analysis {21b}

There is no planned interim analysis.

### Methods for additional analyses (e.g. subgroup analysis) {20b}

There is no planned additional analysis.

### Methods in analysis to handle protocol non-adherence and any statistical methods to handle missing data {20c}

All treatment evaluations will be performed on the principle of intention-to-treat (ITT), using the observed data collected from all randomised participants. We do not anticipate a large number of participants with missing data due to the nature of the study procedures. Cases will be excluded for analyses where data are missing. Reasons for missing data will be described if known.

### Plans to give access to the full protocol, participant-level data and statistical code {31c}

Anonymised data will be made available to other researchers on request for future research in accordance with the University of Auckland data governance policy.

## Oversight and monitoring

### Composition of the coordinating centre and trial steering committee {5d}

The trial steering committee comprises the authors and a clinical research assistant. The trial steering committee will meet monthly to review processes.

### Composition of the data monitoring committee, its role and reporting structure {21a}

The proposed study does not meet the Ellenburg et al. [28] criteria for deciding whether a Data Safety and Monitoring Committee (DMC) needs to be established for a trial. Consequently, a DMC will not be established for the trial.

### Adverse event reporting and harm {22}

This is a very-low-risk study for participants. Participants are able to withdraw from the study at any time and return to their existing hearing aids with their primary audiology provider.

### Frequency and plans for auditing trial conduct {23}

The study team will meet monthly to review all aspects of the study.

### Plans for communicating important protocol amendments to relevant parties (e.g. trial participants, ethical committees) {25}

Any changes to the study protocols will require review by the New Zealand Government Health and Disability Ethics Committee.

### Dissemination plans {31a}

The final study outcomes will be prepared for publication and dissemination. Participants will have access to an overview of the results.

## Discussion

This paper described a protocol for assessing clinically significant differences in hearing aid settings and outcomes between in-clinic and remote hearing aid fittings. Current best practices for audiology require REMs as the gold standard for hearing aid verification but these cannot yet be performed through teleaudiology without the use of specialised equipment and facilitators. If found to be effective, the use of remote fit hearing aids have the potential to significantly increase access to hearing health care services, particularly in hard-to-reach communities.

## Trial status

Protocol version 1.0, 16 May 2023. Participant recruitment has not yet commenced and is expected to be completed by 30 August 2023.

### Supplementary Information

**Supplementary Material 1.**

**Supplementary Material 2.**

## Data Availability

Data and materials will be available to the research team.

## Abbreviations

4FREAR: Four-frequency rear-ear aided response
APHAB: Abbreviated Profile of Hearing Aid Benefit
HHIA: Hearing Handicap Inventory for Adults
NAL-NL2: National Acoustics Laboratory Non-Linear 2 prescription
PMM: Probe-microphone measures
QOL: Quality of Life
QSIN: Quick Speech in Noise
REAR: Real-ear aided response
REM: Real-ear measurement
SII: Speech Intelligibility Index
WHO: World Health Organization
