# Supplementary material for: Remote or in-clinic? The effect of service delivery mode on hearing aid output: study protocol for a double-blinded, randomised trial in adults with mild to moderate sensorineural hearing loss
Source: Trials. 2024 Apr 12;25:256. doi: 10.1186/s13063-024-08068-y (PMC11010415; doi:10.1186/s13063-024-08068-y)
Supplement: Supplementary file 1 — Supplementary Material 1. [file 13063_2024_8068_MOESM1_ESM.pdf]

THE UNIVERSITY OF AUCKLAND
Te Whare Wānanga o Tāmaki Makaurau
NEW ZEALAND

MEDICAL AND HEALTH SCIENCES
SCHOOL OF POPULATION HEALTH

CONSENT FORM

Can the internet be used to fit hearing aids?

- I have read and understood the Participant Information Sheet.
- I have been given sufficient time to consider whether to participant in this study.
- I have had the chance to ask questions and am satisfied with the answers given to me.
- I understand that taking part in this study is voluntary (my choice) and that I may withdraw from the study at any time.
- I understand that whether I take part or not, it will not influence the on-going treatment for hearing loss from my audiologist
- I understand that my participation in this study is confidential and that no information which could identify me personally will be used in any reports on this study.
- I understand that data will be kept indefinitely on a password protected computer, with no identifiable information.
- I understand the time commitments required for this study.
- I know whom to contact if I have any questions about the study in general.
- I wish to receive a summary of the results from the study and understand that there may be a delay between data collection and the publication of the research results.
    - Please circle: Yes No
- I wish to be put into the draw to win an iPad
    - Please circle: Yes No

If you would like to see the summary report or go into the draw to win the iPad, please provide a contact email address or phone number below:

Email or phone number: ____

Please tick to show you consent to the above [ ]

Declaration by participant: I hereby consent to take part in this study

Participant's name: ____

Signature: ____

Date: ____
